# *Anaerobiospirillum succiniciproducens* Community‐Acquired Pneumonia With Bacteraemia in an Immunocompetent Individual

**DOI:** 10.1155/crdi/9072721

**Published:** 2025-12-09

**Authors:** Thomas Ledger, Varun Moorthy, Dean Panos, Katerina D. Arvanitakis, Ravindra Dotel

**Affiliations:** ^1^ Department of Infectious Diseases, Blacktown Hospital, Western Sydney Local Health District, Blacktown, New South Wales, Australia; ^2^ Faculty of Medicine, University of New South Wales, Kensington, New South Wales, Australia, unsw.edu.au

**Keywords:** *Anaerobiospirillum succiniciproducens*, infection and inflammation, pneumonia

## Abstract

An independent 69‐year‐old diagnosed with community‐acquired pneumonia presented with *Anaerobiospirillum succiniciproducens* bacteraemia, initially identified by matrix‐assisted laser desorption ionization–time of flight mass spectrometry. A rarely reported cause of pneumonia and bacteraemia, it is considered a zoonotic bacterium from cats and dogs. She was treated successfully with piperacillin/tazobactam and amoxicillin/clavulanic acid.

## 1. Case Report

A 69‐year‐old female patient presented to our hospital following a syncopal event at home associated with lethargy and malaise. She reported fevers, chills, rigours and coryzal symptoms for 7 days, 2 days of loose stool, and a cough intermittently productive of yellow sputum. She denied haemoptysis, shortness of breath, palpitations or exposure to sick contacts. She was not experiencing any urinary symptoms. She reported a self‐resolving episode of epistaxis.

She was a current cigarette smoker with a 39‐pack‐year history and did not drink alcohol. At home, she lived independently and without support with her husband, a dog and a cat and had an exercise tolerance of 10–30 m. She was retired and had previously worked as a nurse. She had travelled to the Philippines 7 months prior.

She had a history of chronic obstructive pulmonary disease and did not report taking any regular puffers nor having past infective exacerbations. She took linagliptin and supplemental insulin for type 2 diabetes, which was complicated by diabetic nephropathy with stage IV chronic kidney disease and a baseline estimated glomerular filtration rate (eGFR) of 25 mL/min/1.73 m^2^L. She took furosemide 125 mg daily and spironolactone 50 mg daily for peripheral oedema.

Other notable medications included warfarin for an unprovoked left lobe segmental pulmonary embolism diagnosed 9 months prior, levothyroxine for hypothyroidism, and had a past history of entecavir use for chronic e antigen negative hepatitis B infection. She had took pantoprazole for reflux, with a recent surveillance endoscopy and gastroscopy demonstrating angiodysplasia of the gastric curvature, duodenum, ascending colon and cecum.

On examination, she had a Glasgow Coma Scale (GCS) of 15. Her blood pressure was 83/50 mmHg, heart rate 75/min, respiratory rate 21/min and oxygen saturation 95% on room air. The temperature was 35.6°C. She had bilateral basal crackles on auscultation, and right iliac fossa tenderness. Her heart sounds were dual with no murmurs. No musculoskeletal injuries were identified arising from the falls.

Blood tests showed leucocytosis, with an elevated C reactive protein of 376 mg/L. An acute kidney injury was seen, with eGFR to 14 mL/min/1.73 compared to the pre‐morbid eGFR of 20–24 mL/min/1.73 m^2^. Her blood sugar level was 22.8 mmol/L (normal random 4–20 mmol/L) and ketones 1.0 mmol/L (normal 0 mmol/L), with an HbA1c of 10.7%. INR was elevated at 5.5 (Table 1).

**Table 1:** Blood test results on presentation.

Test	Result	Normal range
White cell count	10.5 × 10^9^/L	4–10 × 10^9^/L
Neutrophils	8.7 × 10^9^/L	2–8 × 10^9^/L
Lymphocytes	0.5 × 10^9^/L	1–4 × 10^9^/L
Monocytes	1.0 × 10^9^/L	0.2–1 × 10^9^/L
Eosinophils	0.1 × 10^9^/L	0.0–0.5 × 10^9^/L
Haemoglobin	69 g/L	115–160 g/L
MCV	78 fL	82–98 fL
Platelet count	329 × 10^9^/L	150–450 × 10^9^/L
Venous lactate	2.7 mmol/L	< 2 mmol/L
Albumin	19 g/L	30–44 g/L
Bilirubin	7 μmol/L	< 21 μmol/L
Alanine aminotransferase	12 IU/L	6–36 IU/L
Aspartate transaminase	18 IU/L	10–28 IU/L
Alkaline phosphatase	88 IU/L	32–100 IU/L
Lipase	82 μ/L	10–60 U/L
Sodium	133 mmol/L	135–145 mmol/L
Potassium	4.9 mmol/L	3.5–5.0 mmol/L
Creatinine	257 μmol/L	45–90 μmol/L
eGFR	16 mL/min/1.73 m^2^	> 90 mL/min/1.73 m^2^
INR	5.5	< 1.5
C reactive protein	376 mg/L	< 4 mg/L

A chest x‐ray (Figure 1(a)) demonstrated bilateral lower lobe consolidation. A CT scan of chest and abdomen without contrast confirmed extensive consolidation in both lower lobes (Figure 1(b)), with associated small reactive mediastinal and pulmonary hilar lymph nodes and background emphysematous changes and bronchiectasis.

Figure 1Chest x‐ray on admission showing bilateral lower lobe consolidation (a), and axial slice of a CT chest on admission (b) showing bilateral consolidation and bronchiectatic changes.

**Figure:**
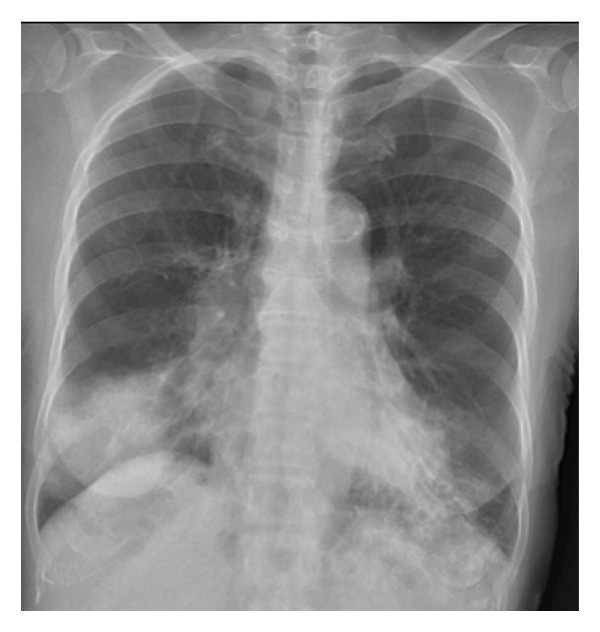
(a)

**Figure:**
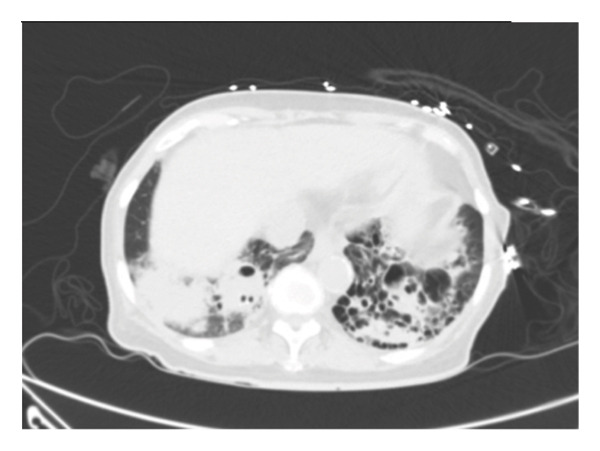
(b)

An anaerobic blood culture bottle (Bactec Lytic, Bactec, Canada) in a paired set of blood cultures, became positive after 2 days, and Gram staining showed spiral‐shaped Gram‐negative organisms. The organism was identified as *Anaerobiospirillum succiniciproducens* by matrix‐assisted laser desorption ionization–time of flight mass spectrometry (MALDI‐TOF MS) using the Vitek MS version MS‐CE CLI 2.0.0 on intact cells without extraction (bioMérieux Inc., Durham, NC). For reasons of diagnostic stewardship, antibiotic susceptibility testing is not routinely performed for anaerobes in our laboratory, and, given the patient’s improvement, none was requested. A microbiological workup for other pathogens associated with community‐acquired pneumonia was negative, including pneumococcal and legionella urinary antigens, and a respiratory viral multiplex PCR was including testing for COVID‐19, influenza, human metapneumovirus, RSV, rhinovirus, enterovirus, parainfluenza, and adenovirus. The patient was unable to expectorate sputum for analysis.

She was diagnosed with *A*. *succiniproducens* bacteraemia with associated pneumonia, with a clinically compatible illness and a clear epidemiological exposure via her pets. After resuscitation in the emergency department, she was admitted to the intensive care unit for vasopressor support for 24 h. Her supratherapeutic INR was associated with an episode of epistaxis and reversed with phytomenadione. She was treated with 7 days of intravenous piperacillin/tazobactam, and a further 7 days of oral amoxicillin/clavulanate 875/125 mg twice daily, and improved to her baseline health.

## 2. Discussion

A. succiniciproducens is an anaerobe rarely reported as the cause of human disease. When it is reported, it is generally as a cause of diarrhoea, and rarely bacteraemia, and generally in immunocompromised hosts [1]. This case reports adds to the literature by describing a predominantly respiratory presentation and sepsis in an immunocompetent host, and further illustrates the global distribution of this organism by describing a case in the Oceania region.

Anaerobiospirillum was first identified in 1976 in Beagle dogs [2]. It is part of the normal intestinal flora of cats and dogs [1] and therefore speculated to be a zoonotic illness when it affects humans. It has not been identified in the health human gut flora and human to human transmission has not been documented; it is proposed that gut translocation following exposure to a zoonotic carrier may represent the mechanism of bacteraemia [1].

A review of literature in 2011 by Kelesidis analysed 33 cases of *A. succiniciproducens* bacteraemia, seen amongst 56 total published cases of infection, of which only 4 (7.1%) were in our Oceania region. The characteristics of patients with *A. succiniciproducens* bacteraemia (*n* = 33) are shown in Table 2.

**Table 2:** Characteristics of 33 patients with *A. succiniciproducens* bacteraemia [1].

Statistics	Value
Age (mean, range)	54.1 (1 months–90 years)
Gender (*n*, %)	Male (19, 57.6%)
Risk factors	
Chronic alcoholism	11 (39%)
Malignancy	10 (36%)
Atherosclerosis	8 (29%)
Recent surgery	4 (14%)
Liver disease	3 (11%)
Insulin‐dependent diabetes	3 (11%)
Poor dentition	3 (11%)
Animal exposure	3 (11%)
Chemotherapy, splenectomy, or AIDS	2 (7%)

The case we present here is distinguished from published cases by the predominantly respiratory symptoms leading to presentation, reported only in one other case, also in Australia [3]. In contrast, gastrointestinal signs and symptoms were reported in 17 of 24 patients with bacteraemia who had symptoms [1]. MALDI‐TOF has previously been used to identify *A. succiniciproducens*, [4], highlighting its usefulness in detecting this uncommon pathogen [5].

*A. succiniciproducens* is generally reported susceptible to a large variety of antimicrobials, including, notably, amoxicillin, amoxicillin‐clavulanic acid, and cefuroxime, which form a standard part of pneumonia treatment guidelines [1, 6, 7] Notably, they tend to be resistant to metronidazole and clindamycin, a more commonly used anaerobic antibiotic [1]. There are no standard breakpoints are available for this rarely reported infection in the standard reference European Committee for Antimicrobial Susceptibility Testing (EUCAST) or Clinical and Laboratory Standards Institute (CLSI) guidelines.

## Consent

The authors declare that appropriate written informed consent was obtained for the publication of this manuscript and accompanying images.

## Conflicts of Interest

The authors declare no conflicts of interest.

## Author Contributions

All authors contributed to the writing of the manuscript.

Thomas Ledger contributed to the primary manuscript, review, and submission.

Varun Moorthy and Dean Panos contributed to writing the primary manuscript and review.

Ravindra Dotel provided significant input into the manuscript and review.

## Funding

The authors received no specific funding for this work.
